# Specificity of DNA-binding by the FAX-1 and NHR-67 nuclear receptors of *Caenorhabditis elegans *is partially mediated via a subclass-specific P-box residue

**DOI:** 10.1186/1471-2199-9-2

**Published:** 2008-01-07

**Authors:** Stephen D DeMeo, Rebecca M Lombel, Melissa Cronin, Eric L Smith, Danielle R Snowflack, Kristy Reinert, Sheila Clever, Bruce Wightman

**Affiliations:** 1 Biology Department, Muhlenberg College, Allentown, PA 18104, USA; 2 Current address : Philadelphia College of Osteopathic Medicine, Philadelphia, PA 19131, USA; 3 Current address : Department of Pediatrics, University of Michigan Health System, Ann Arbor, MI 48109, USA; 4 Current address : Department of Genetics and Genomic Sciences, Mount Sinai School of Medicine, New York, NY 10029, USA; 5 Current address : Department of Molecular Biology, Princeton University, Princeton, NJ 08544, USA; 6 Current address : School of Medicine, University of Pennsylvania, Philadelphia, PA 19104, USA

## Abstract

**Background:**

The nuclear receptors of the NR2E class play important roles in pattern formation and nervous system development. Based on a phylogenetic analysis of DNA-binding domains, we define two conserved groups of orthologous NR2E genes: the NR2E1 subclass, which includes *C. elegans nhr-67, Drosophila tailless *and *dissatisfaction*, and vertebrate Tlx (NR2E2, NR2E4, NR2E1), and the NR2E3 subclass, which includes *C. elegans fax-1 *and vertebrate PNR (NR2E5, NR2E3). PNR and Tll nuclear receptors have been shown to bind the hexamer half-site AAGTCA, instead of the hexamer AGGTCA recognized by most other nuclear receptors, suggesting unique DNA-binding properties for NR2E class members.

**Results:**

We show that NR2E3 subclass member FAX-1, unlike NHR-67 and other NR2E1 subclass members, binds to hexamer half-sites with relaxed specificity: it will bind hexamers with the sequence ANGTCA, although it prefers a purine to a pyrimidine at the second position. We use site-directed mutagenesis to demonstrate that the difference between FAX-1 and NHR-67 binding preference is partially mediated by a conserved subclass-specific asparagine or aspartate residue at position 19 of the DNA-binding domain. This amino acid position is part of the "P box" that plays a critical role in defining binding site specificity and has been shown to make hydrogen-bond contacts to the second position of the hexamer in co-crystal structures for other nuclear receptors. The relaxed specificity allows FAX-1 to bind a much larger repertoire of half-sites than NHR-67. While NR2E1 class proteins bind both monomeric and dimeric sites, the NR2E3 class proteins bind only dimeric sites. The presence of a single strong site adjacent to a very weak site allows dimeric FAX-1 binding, further increasing the number of dimeric binding sites to which FAX-1 may bind *in vivo*.

**Conclusion:**

These findings identify subclass-specific DNA-binding specificities and dimerization properties for the NR2E1 and NR2E3 subclasses. For the NR2E1 protein NHR-67, Asp-19 permits binding to AAGTCA half-sites, while Asn-19 permits binding to AGGTCA half-sites. The apparent conservation of DNA-binding properties between vertebrate and nematode NR2E receptors allows for the possibility of evolutionarily-conserved regulatory patterns.

## Background

The nuclear receptors are a class of transcriptional regulatory proteins that function in physiology and development in animals [1-3]. They are conserved from sponges to mammals [4,5], but this class of proteins has seen significant amplification in the number of genes and elaboration of the amino acid sequences in nematode species [6-8]: while humans have 48 predicted nuclear receptor genes and *Drosophila melanogaster *has 21, *Caenorhabditis elegans *and *C. briggsae *each have over 250. Most nuclear receptors have two major conserved functional domains: the DNA-binding domain (DBD) and the ligand-binding domain (LBD). These two domains are normally found in that order from N-terminus to C-terminus, although there are unorthodox nuclear receptors that have no apparent LBD, such as KNIRPS and ODR-7 [9,10], and some that have no DBD, such as DAX1 and SHP [11,12].

**Figure 1:**
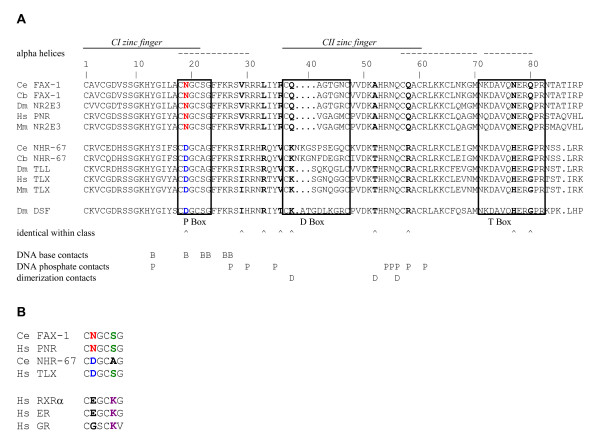
**DBD amino acid comparison**. A. Alignment of the amino acid sequence of NR2E DNA-binding domains from NR2E1 and NR2E3 subclasses. The sequences were aligned as described in Methods. Potential amino acid residues that might make DNA base contacts (B), phosphate contacts (P), and dimerization contracts (D) were inferred from structural studies on RXR/RAR heterodimer recognition of DR1 sites and similar studies [35, 36]. B. Comparison of P box sequences of NR2E1 and NR2E3 subclasses to other *C. elegans *nuclear receptors examined in this study and other representative nuclear receptors from humans. Species abbreviations: Ce, *Caenorhabditis elegans; *Cb, *Caenorhabditis briggsae; *Dm, *Drosophila melanogaster; *Hs, *Homo sapiens; *Mm, *Mus musculus*.

The nuclear receptor DBD is responsible for binding to a specific DNA response element (NRE) in the promoters of target genes, relatively weak dimerization contacts, and nuclear localization [3]. The DBD consists of two C-4 zinc finger structures at its N-terminal portion and a C-terminal extension (CTE), which is sometimes grouped with the "hinge region" between the DBD and LBD (Fig. 1). Two key regions in the DBD have been shown to play major roles in NRE binding specificity by making direct and water-mediated hydrogen bond contacts with specific DNA bases. The P box consists of six conserved amino acids in an alpha-helical region that begins in the C-terminal portion of the first zinc finger [13-15]. The A box is a poorly-conserved C-terminal portion of the CTE, and is thought to play a role in modulating DNA specificity, although this function is not as well understood [16]. A few other conserved amino acids C-terminal to the P box also contact DNA bases, and several other amino acids spread throughout the DBD make contact with the DNA phosphodiester backbone. Two other regions of the DBD play key roles in dimerization, although some nuclear receptors are unable to dimerize without the strong dimerization function of the LBD. In the DBD, the D box, located in the N-terminal portion of the second zinc finger, and the alpha-helical T box of the CTE have been demonstrated to play direct roles in dimerization between DBD partners [14,17]. In most cases, nuclear receptors bind to cognate NRE's via the DBD, however there are examples of nuclear receptors that can provide some regulatory function without binding to DNA through their DBD [18].

The LBD was named based on its function in binding lipophilic hormones in vertebrates and insects [1]. However, some nuclear receptors, such as Nurr1, are clearly ligand-independent [19] and many more are believed to be (the so-called orphan receptors). The LBD functions in binding coactivator and corepressor proteins, which in turn interact with other transcription factors or modulate chromatin structure in order to affect changes in transcriptional rates of target genes. The LBD also plays a major role in dimerization and nuclear localization [3].

The NR2E subclass of nuclear receptors is evolutionarily conserved in both vertebrates and invertebrates. The founding member, *tailless *(Tll; NR2E2), was first identified by a genetic approach in *Drosophila*, where it functions in anterior-posterior pattern formation and central nervous system development [20,21]. The vertebrate ortholog, Tlx (NR2E1), functions in limbic system and adult neural stem cell development [22,23]. The *C. elegans *ortholog, *nhr-67*, appears to function in various aspects of hypodermal and neural development and organogenesis [8]. The *Drosophila *gene *dissatisfaction *(Dsf; NR2E4) encodes a possible Tll paralog (or alternatively a distinct ancestral NR2E member that was subsequently lost in nematode and vertebrate lineages [4]) that functions in the development of sex-specific neural and muscular features [24]. The final NR2E gene member is represented by PNR (NR2E3) in vertebrates, and *fax-1 *(NR2E5) in *C. elegans*. In humans and mice, PNR functions in specifying photoreceptor identity in the retina [25-28], while in *C. elegans*, *fax-1 *functions in specification of interneuron identity [29-31]. One phylogenetic analysis of PNR and *fax-1 *family members from different species suggested a possible early divergence between the two genes, with subsequent loss of *fax-1 *from vertebrate lineages and loss of PNR from nematode lineages [4].

The identification of DNA binding specificities for different nuclear receptors has been fruitful in predicting downstream target genes and in understanding the molecular logic of how DBD amino acid sidechains interact with NRE bases and the phosphodiester backbone. Studies on *Drosophila *Tll and vertebrate Tlx binding specificities suggested that the NR2E receptors bind to half-sites with a distinctive sequence, AAGTCA, instead of the more common NRE half-site AGGTCA [32,33]. Dsf also preferentially binds AAGTCA half-sites, and like Tll and Tlx can bind either as a monomer or dimer [34]. Initial DNA-binding studies showed that PNR also bound to AAGTCA half-sites, but only as a dimer bound to directly-repeated half-sites separated by a single base-pair (DR1 sites) [25]. Thus an adenine residue at the second position of the NRE half-site has been suggested to be a defining feature of NR2E half-site specificity.

Examination of the P boxes of NR2E subclass members suggested a possible amino acid that could mediate the difference in specificity between this subclass and other nuclear receptors. The nuclear receptors RXR, NGF1-B, RAR, RevErb, EcR and USP all preferentially bind AGGTCA half-sites and have P boxes with the amino acid sequence CEGCKG [3]. In contrast, the NR2E class members all have the P box sequence C(D/N)GC(S/A)G. The fifth amino acid in the P box, lysine-22 (numbering scheme begins with 1 designated as the first cysteine in the DBD), has been shown to make direct hydrogen bond contacts to the base at the second position of the NRE in co-crystal structures for nuclear receptors that bind the AGGTCA half-site [3,35]. Therefore, it has been suggested that lysine-22 may confer specificity for binding a guanine residue at NRE position two. In contrast, a serine or alanine at DBD position 22, as found in the NR2E nuclear receptors, may confer specificity for binding an adenine base at NRE position two [3].

A structural study that examined RXR-RAR binding to DR1 dimeric AGGTCA sites identified the amino acids that make direct and water-mediated contacts to hexamer bases [35]. Guanine-2 of the half-site was contacted by Tyr-13, Glu-19, and Lys-22 of RXR and RAR. These amino acids also made contact to other half-site bases, and other amino acids in the DBD and CTE made direct and water-mediated contacts to other half-sites bases and to the phosphodiester backbone. Tyr-13 is common to all NR2E DBD's (Fig. 1) and many other nuclear receptors that bind AGGTCA half-sites, thus it is unlikely to mediate differences in NRE binding specificity. Given these data, the amino acid present at DBD position 19, in addition to the previously mentioned position 22, could mediate binding specificities at position two of the NRE half-site.

An understanding of DNA binding specificity of the NR2E class of nuclear receptors requires a comparison of the DNA-binding properties of NR2E1 (Tll/Tlx/NHR-67) and NR2E3 (PNR/FAX-1) subclass members. We have examined the DNA binding properties of the FAX-1 nuclear receptor of *C. elegans*, a PNR ortholog, and compared them to the NHR-67 nuclear receptor of *C. elegans*, a Tll ortholog. We find that unlike NR2E1 subclass members, FAX-1 will bind half-sites with the sequence ANGTCA and does not bind to monomeric sites. We tested whether the conserved Asn-19 in NR2E3 subclass members was a key determiner of specificity for binding to NRE half-site position two. Changing Asp-19 of NHR-67 to Asn-19 was sufficient to change the binding preference of NHR-67 from AAGTCA half-sites to AGGTCA half-sites. In contrast, changing Asn-19 of FAX-1 to Asp-19 was not sufficient to change the DNA-binding preference, suggesting that the role of DBD position 19 in binding specificity is context-dependent.

## Results

### FAX-1 and orthologs have a distinct P box

As a first step toward identifying amino acids that might confer subclass-specific DNA binding activities, we aligned amino acid sequences of the DBD's, including the CTE, of FAX-1 and orthologs from vertebrate and invertebrate species and compared them to equivalent sequences from other NR2E class members (Fig. 1). This analysis included five members of the NR2E3 subclass (FAX-1 and orthologs) and six members of the NR2E1 subclass (Tll and orthologs). Due to its similar DNA-binding activity, Dsf (NR2E4) was grouped with the NR2E1 subclass for this analysis. Subclasses and individual members within a subclass differed in the length of the D box. While all NR2E3 subclass members had a D box containing eight amino acids, the NR2E1 subclass members had either nine (vertebrate and *Drosophila *Tll), eleven (Dsf) or twelve (*C. elegans *NHR-67). These differences might contribute to the different dimerization properties of Tll as compared to PNR. With FAX-1 as the reference sequence, all the NR2E members evaluated were identical at 48 of 85 (56%) amino acid residues, as expected for the strong evolutionary conservation observed for nuclear receptor DBD's. The NR2E DBD's had variability within a subclass (for example, DBD position 2 can be Ala, Val, Arg, or Lys without a consistent difference between the NR2E1 and NR2E3 subclasses) at 28 of 85 (33%) residues. In some cases, the variability was limited to amino acids with similar chemical properties. For example, position 16 can be Leu, Tyr or Phe, all of which are hydrophobic residues. The remaining nine amino acid residue positions of the DBD's were identical within a subclass, but different between subclasses. For example, position 29 is a Val in all members of the NR2E3 subclass and Ile in all members of the NR2E1 subclass. This last group identifies the amino acids that might play roles in differing DNA-binding specificity of the NR2E3 subclass as compared to the NR2E1 subclass. Of particular note, position 19, which is a P box residue predicted to contact the DNA, has an Asn in all members of the NR2E3 subclass and an Asp in all members of the NR2E1 subclass.

### FAX-1 binds DR1 sites with relaxed specificity

**Figure 2:**
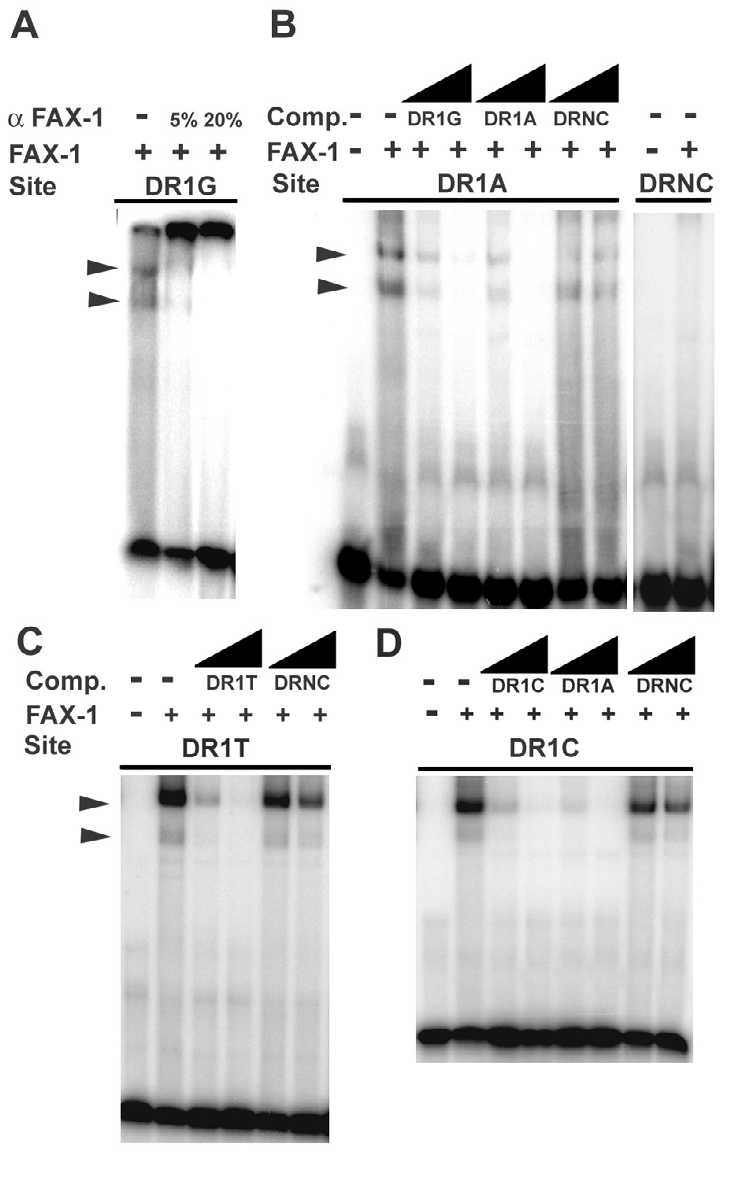
**Binding of FAX-1 to DR1 dimeric sites**. A. EMSA of FAX-1 protein binding to DR1G sequences (AGGTCA direct repeats), showing supershift of bands in 5% and 20% mouse anti-FAX-1 antiserum [31]. B. EMSA of FAX-1 protein binding to DR1A sequences (AAGTCA DR1) and failing to bind to DRNC sequences (AATTCA repeats). Binding could be competed with 10-fold and 100-fold molar excess of unlabeled competitor DR1G and DR1A DNA, but not DRNC DNA (wedges). Similar results (not shown) were obtained using radioactively-labeled DR1G DNA (AGGTCA DR1). C. and D. EMSA of FAX-1 protein binding to DR1T sequences (ATGTCA DR1) and DR1C sequences (ACGTCA DR1), respectively.

**Figure 3:**
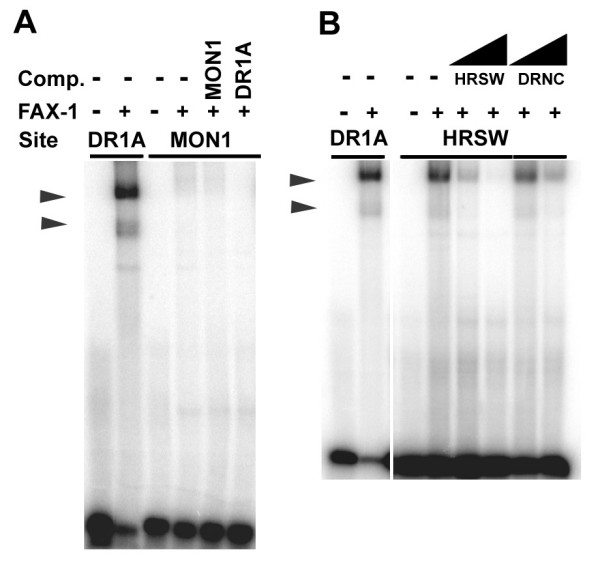
**Binding of FAX-1 to monomeric and heteromeric strong/weak sites**. A. EMSA of FAX-1 protein binding to MON1 sequences (single AAGTCA binding site). Competition with 10-fold molar excess of unlabeled MON1 and DR1A DNA sequences did not reduce non-specific background bands. Similar results (not shown) were obtained with MON2 sequences (single AAGTCA site in different position). B. EMSA of FAX-1 protein binding to HRSW sequences (AAGTCA strong binding site followed by AATTCA weak binding site). Binding could be competed with 10-fold and 100-fold molar excess of unlabeled competitor HRSW DNA, but not DRNC DNA (wedges). Although we obtained strong shifted bands, the proportion of labeled DNA shifted was considerably less than that observed with DR1A sequences (Table 1). We obtained similar results (not shown) with HRWS DNA (AATTCA weak site followed by AAGTCA strong site).

We used the electrophoretic mobility shift assay (EMSA) to test binding of bacterially-expressed and purified FAX-1 protein ([31]; Fig. 2). Binding of FAX-1 protein to NRE-containing DNA (Table 1) resulted in two discrete shifted bands, which were inhibited and super-shifted by the addition of anti-FAX-1 antiserum (Fig. 2A). The relative intensities of these two bands varied somewhat from experiment to experiment. The lower band was neither preferentially reduced by competitor DNA containing a monomeric binding site, nor was it preferentially observed in experiments using a monomeric binding sites (MON1 and MON2; Fig. 3), indicating that the two bands probably represent two different topological states of protein-DNA complexes (or variable presence of a proteolytic fragment) rather than monomeric and dimeric protein-DNA complexes. Because both bands showed similar behavior in competition and supershift experiments, we have summed both bands as being indicative of FAX-1-DNA binding throughout this paper. FAX-1 protein produced no discernible shifted products at these locations when allowed to bind negative control DNA carrying a dimeric AATTCA sequence (DRNC; Fig. 2B).

**Table 1:** DNA sequences tested for NR2E binding activity

**Site Name**	**Sequence**	**% Bound by FAX-1**	**S.D**.
DR1A	**AAGTCAaAAGTCA**	53.3	13.6
DR1G	**AGGTCAaAGGTCA**	42.1	11.8
DR1C	**ACGTCAaACGTCA**	26.1	17.2
DR1T	**ATGTCAaATGTCA**	29.8	1.9
DRNC	**AATTCAaAATTCA**	0.4	0.2
MON1	**AAGTCAaAATTTA**	2.8	0.6
MON2	**AATTTAaAAGTCA**	4.5	1.0
HRSW	**AAGTCAaAATTCA**	11.7	1.0
HRWS	**AATTCAaAAGTCA**	10.5	0.9
DR2A	**AAGTCAaaAAGTCA**	20.7	6.8
DR3A	**AAGTCAaaaAAGTCA**	2.3	0.4

The first column indicates the name used to refer to each sequence in the text. The second column shows the actual sequence tested. The third column indicates the percentage of the labeled DNA that was shifted to high mobility in EMSA experiments using FAX-1 protein. The fourth column indicates standard deviation for each set of EMSA experiments summarized. We evaluated the significance of the data using One-Way ANOVA and Fisher 95% Confidence Intervals for all pairwise comparisons. DR1A and DR1G were not significantly different from each other. DR1A was significantly different from all other sequences tested (p < 0.05). The difference between DR1G and DR1C or DR1T fell just outside the range of statistical significance. Binding data for MON1, MON2, DR3A, and DRNC sites were not significantly different from each. Pairwise differences between HRWS or HRSW and MON1 or MON2 were significant (p < 0.05).

Because the FAX-1 vertebrate ortholog, PNR, has been shown to bind AAGTCA dimeric sites separated by one base-pair (DR1 sites) [25], we first tested whether FAX-1 would display the same binding activity. FAX-1 protein bound to DNA containing AAGTCA dimeric sequences (DR1A; Fig. 2B; Table 1), and this binding could be competed with similar effectiveness by DNA containing AAGTCA DR1A dimers or AGGTCA DR1G dimers, but not negative control AATTCA dimers (DRNC; Fig. 2B). Thus FAX-1 can bind AAGTCA dimers specifically, similar to PNR and Tll.

We tested whether FAX-1 could discriminate among different nucleotides at the second position, as Tll does [32-34]. Unlike Tll, FAX-1 bound to AGGTCA DR1G dimers with avidity similar to that of AAGTCA DR1A dimers (Fig. 2A; Table 1). Furthermore, FAX-1 also bound dimers with pyrimidines in the second position: specific binding, although significantly weaker, was also observed with ACGTCA and ATGTCA dimers (DR1C and DR1T; Fig. 2C–D; Table 1). Unlabeled DR1A DNA competed more effectively for binding to DR1C sites than did unlabeled DR1C DNA (Fig. 2D). Therefore, in contrast to Tll and Tlx, FAX-1 does not discriminate among different nucleotides at the second hexamer position, although it binds purines more strongly than pyrimidines.

### FAX-1 does not bind monomeric hexamers but can bind a weak site adjacent to a strong site

We tested next whether FAX-1 could bind both a single copy of the hexamer and a dimeric copy of the hexamer, like Tll, Tlx, and Dsf, or could bind only a dimeric copy of the hexamer, like PNR. When challenged to bind DNA containing only a monomeric copy of the AAGTCA hexamer (MON1 or MON2), FAX-1 showed negligible binding that was not significantly different from negative controls (DRNC; Fig. 3A; Table 1). Consistent with this result, unlabeled DNA containing monomeric AAGTCA sequences competed very poorly for binding to labeled AAGTCA DR1A or AGGTCA DR1G dimeric sequences (data not shown).

In contrast to the results for monomeric sites, when we placed a predicted weak binding site (AATTCA) in register for DR1 binding with a strong site (AAGTCA), creating heteromeric repeats in either of the two possible orders (HRSW or HRWS), we found weak, but significant, binding by FAX-1 (Fig. 3B; Table 1). This binding was approximately 1/3 as strong as binding to ATGTCA dimers and 1/5 as strong as binding to AAGTCA dimers (Table 1), and significantly different from DRNC controls. These data are consistent with a model where FAX-1 binds as an obligate dimer, but binding to a strong site can facilitate binding to an adjacent weaker site through cooperativity.

### FAX-1 can bind DR2 sites but not DR3 sites

**Figure 4:**
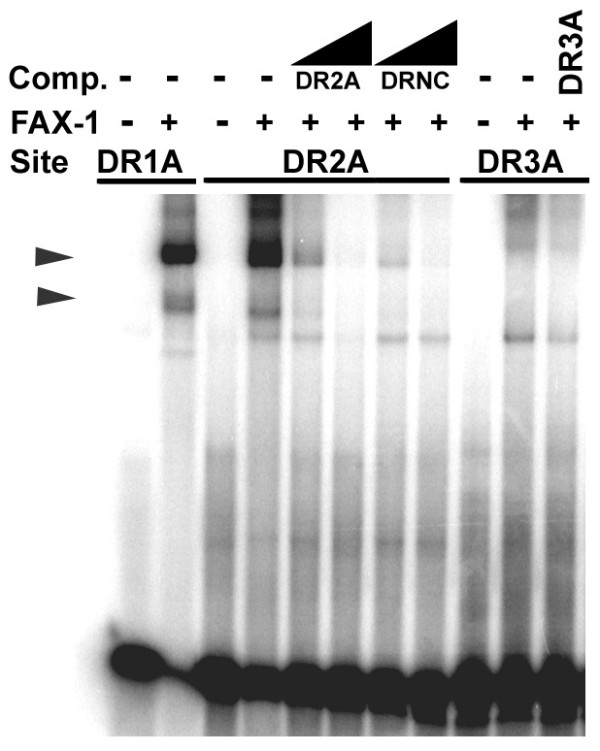
**Binding of FAX-1 to DR2 and DR3 dimeric sites**. EMSA of FAX-1 protein binding to DR2A and DR3A sequences (dimeric AAGTCA binding sites separated by two base-pairs and three base-pairs, respectively). Competition experiments with DR2A, DR3A, and DR1A are shown at 10-fold and 100-fold molar excess (wedges) or 10-fold molar excess only (DR3A). We observed additional shifted species in this experiment, however these bands could not be competed with equivalent unlabeled oligonucleotides.

Nuclear receptor dimerization confers limitations on the amount of separation between the two hexamer half-sites that will be tolerated by a given protein [3]. Our data show that FAX-1 can bind DR1 sites, which separate the two hexamers with a single base-pair, similar to Tll and PNR. We tested whether increasing the separation between the two hexamers would influence the strength of FAX-1 binding. When AAGTCA hexamers that are separated by two base-pairs (DR2 sites; DR2A) were tested, FAX-1 could bind these sequences, albeit not as strongly as DR1 sites (Fig. 4). The strength of binding, as measured by EMSA, was similar to the strength of binding to ACGTCA dimers and about half that observed with AAGTCA dimers (Table 1). These data show that FAX-1 prefers DR1 sites, but can also bind DR2 sites.

When we further increased the separation of the two AAGTCA hexamers to three base-pairs (DR3 sites; DR3A), we were no longer able to detect binding by FAX-1 (Fig. 4). These data demonstrate that FAX-1 dimerization requires relatively close proximity of the two hexamers, and also serve to reinforce the conclusion that FAX-1 is unable to bind as a monomer.

### DBD amino acid 19 partially mediates DNA-binding specificity in FAX-1 and NHR-67

**Figure 5:**
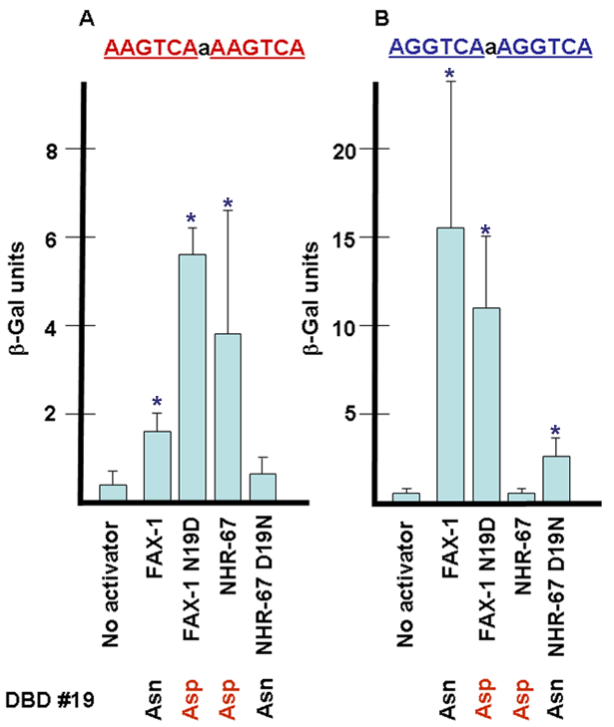
**One-hybrid analysis of FAX-1 and NHR-67 DNA binding activity**. A. β-galactosidase activity in one-hybrid experiments for yeast containing DR1A binding sites. All strains contained a reporter plasmid derived from pLacZi that contained a single DR1A binding site upstream of the *lacZ *gene. Each strain included either no activator or a fusion construct containing a nematode nuclear receptor DBD fused to the yeast GAL4 activation domain. B. β-galactosidase activity for yeast containing DR1G binding sites. Error bars show standard deviations. Asterisks indicate results that are significantly different than no activator control by student's t-test (p < 0.05). The difference between the FAX-1 and FAX-1 N19D mutant activators on DR1A sites is also statistically significant. We performed equivalent experiments using strains containing negative control DRNC sites and activator constructs. These strains did not show β-gal activity relative to controls that had no activator (data not shown). We performed equivalent experiments using strains containing MON1 sites and HRWS sites (both AAGTCA monomers) and a FAX-1 DBD activator, which also did not show β-gal activity relative to controls (data not shown).

In order to investigate differences between NR2E1 and NR2E3 subclass DNA-binding properties and to allow for mutagenesis of the subclass-specific DBD amino acid 19, we constructed *fax-1 DBD::GAL4 activation domain *and *nhr-67 DBD::GAL4 activation domain *fusion genes and tested their ability to activate transcription from promoters that carry different potential binding sites using the yeast one-hybrid system [16]. As expected from our EMSA analysis, the FAX-1 DBD conferred significant β-galactosidase activity in one-hybrid studies with both AAGTCA DR1A and AGGTCA DR1G dimers in this assay (Fig. 5), but did not with an AAGTCA monomer (data not shown). Also consistent with previous results for the NR2E1 subclass [32-35], the NHR-67 DBD conferred significant β-galactosidase activity with AAGTCA DR1A dimers (Fig. 5A), but not with AGGTCA DR1G dimers (Fig. 5B). These data demonstrate that for FAX-1 and NHR-67, the DBD alone is sufficient to confer at least some half-site recognition and dimerization properties, and that the modality of binding inferred from our one-hybrid experiments yields data that are consistent with our direct assessment of FAX-1 binding activity by EMSA and the published direct assessment of Tll and Tlx binding [32,33]. Therefore, testing DNA binding of mutated versions of FAX-1 and NHR-67 by one-hybrid should yield data that are indicative of altered binding preference *in vitro*.

Curiously, binding of FAX-1 to AGGTCA DR1G dimers gave much stronger quantitative results than AAGTCA DR1A dimers in this assay, in contrast to the expected quantitative results from our EMSA studies, which showed similar binding to both dimeric sites. This difference may be due to FAX-1 protein sequences that were not present in the FAX-1 DBD construct or due to artifacts related to DNA sequence context in the reporter constructs or yeast cellular environment. Given this result, and because of the indirect nature of the one-hybrid assay, we have not drawn conclusions about relative binding strength based on quantitative comparisons using this assay.

Asn-19 of the FAX-1 DBD is the only amino acid in the P box that is identical in all known NR2E3 subclass members and is different from the Asp-19 that is consistently found in all known NR2E1 subclass members, including NHR-67 (Fig. 1). Because NHR-67 and Tll discriminate between NRE half-sites that differ at the second position and FAX-1 does not, we hypothesized that the difference between the different DNA-binding specificities might depend on which amino acid is present at position 19. This hypothesis is supported by structural data that show water-mediated hydrogen-bond contacts between this amino acid and the second position of the NRE for nuclear receptors that bind DR1 dimers [35].

To test this hypothesis, we mutated the FAX-1 DBD in the *fax-1 DBD::GAL4 activation domain *one-hybrid construct from the wild-type codon for Asn to a codon for Asp. Mutating the FAX-1 DBD from Asn-19 to Asp-19 significantly increased the β-galactosidase activity observed in one-hybrid studies with AAGTCA DR1A sites approximately 3.7-fold (Fig. 5A). However, the mutation did not significantly decrease or abolish the activity observed with AGGTCA DR1G sites (Fig. 5B). Therefore, converting Asn-19 to Asp-19 in the context of a FAX-1 DBD was not sufficient to change the inferred binding specificity to that of NHR-67. Due to the caveats in interpreting quantitative results from one-hybrid studies, it is unclear whether the Asn to Asp change results in increased binding of FAX-1 to AAGTCA DR1A sites, although our results are consistent with that possibility.

More convincing results were obtained when we performed the reciprocal experiment in which the NHR-67 DBD was mutated from Asp-19 to Asn-19. This mutation eliminated detectable β-galactosidase activity in one-hybrid studies with AAGTCA DR1A sites (Fig. 5A), and produced significant β-galactosidase activity in one-hybrid studies with AGGTCA DR1G sites (Fig. 5B). From these data, we infer that while wild-type NHR-67 with Asp-19 can bind AAGTCA sites but not AGGTCA sites, mutant NHR-67 with Asn-19 can bind AGGTCA sites but not AAGTCA sites. Thus this single amino acid change had the effect of completely reversing the modality of NHR-67 binding activity. In this context, an Asn-19 changed the preference of binding at NRE position two from adenine to guanine. These results indicate that the amino acid present at position 19 in the DBD of NHR-67, in particular, and perhaps NR2E nuclear receptors in general, is a mediator of DNA binding specificity.

## Discussion

Our findings demonstrate that FAX-1 binds ANGTCA half-sites that are separated by one base-pair and can bind AAGTCA half-sites that are separated by two base-pairs. This contrasts it with Tll and NHR-67, which display a strong preference for AAGTCA half-sites. FAX-1 binds more strongly to sites that have a purine at the second position on this strand, but does not distinguish between AAGTCA and AGGTCA. Amino acid 22 of the DBD is known to contact position 2 of the NRE, and it has been proposed that having a Ser or Ala at this position rather than a Lys may allow for recognition of AAGTCA instead of AGGTCA half-sites [3]. All known NR2E class receptors, including FAX-1 and NHR-67, have a Ser or Ala at position 22, suggesting that they may be able to bind the AAGTCA sequence via this amino acid residue. However, we have shown that a difference in DNA-binding specificity exists between members of the NR2E1 and NR2E3 subclasses. In the context of an NHR-67 DBD, the ability to bind guanine at the second position of the NRE hexamer is conferred, in part, by the Asn-19 found in all NR2E3 members, while the ability to bind adenine at the second position of the NRE hexamer is conferred by the Asp-19 found in NR2E1 members.

Despite the important role of DBD position 19, it is also clear that the amino acid at this position is not sufficient to confer a particular mode of binding specificity. Other amino acids in the DBD, perhaps others that are consistently different between classes, must also contribute to NRE binding specificity. An example of another amino acid residue that could participate in subclass specificity is position 58 of the DBD, which is consistently Gln in NR2E3 and Arg in NR2E1 and is predicted to make phosphate backbone contacts with the DNA (Fig. 1).

By analogy to co-crystal structures for RAR-RXR heterodimers bound to DR1 sites [35], Asn-19 would be predicted to make water-mediated hydrogen bonds to the pyrimidine bases on the strand complementary to the adenine or guanine at the second position of the NRE. The implication of this comparison is that Asp binds most favorably to thymine on the complementary strand, while Asn binds favorably to cytosine and may be able to interact favorably with any of the four bases, depending on context. Asn can make a greater variety of H-bond contacts than Asp due to the presence of the amide donor and acceptor in the R group of Asn in comparison to the acceptor-only carboxyl of Asp. This greater range of potential H-bonding may be a contributor to the relaxed specificity observed for NR2E3 subclass DNA binding.

Inferences drawn from our data, the co-crystal structure of nuclear receptors that bind DR1 sites, and general data on nuclear receptor-NRE interactions allow us to develop a model for the remainder of FAX-1-NRE interactions. In co-crystal structures, the last four bases of the hexamer NRE half site, GTCA, are contacted by hydrogen bonds from DBD amino acid 26 and 27, usually LysArg or ArgArg [3]. In all FAX-1 and Tll subclass members, these positions are LysArg. Therefore, it seems reasonable to suggest that the LysArg dipeptide sequence of FAX-1 contacts the last four base-pairs of the NRE. However, these amino acids are apparently not sufficient to confer this binding preference since the steroid receptors also have the LysArg sequence, yet bind a very different half-site [3]. These and other observations raise the important caveat that the geometry of nuclear receptor- DNA binding can vary among classes [1,17,32,36]; therefore the implications of our study may be limited to the NR2E class.

We have not explored specificity at the first position of the NRE half-site, which might also be somewhat degenerate. Based on the inference drawn from the RAR-RXR co-crystal on DR1 sites, we would expect that the adenine at the first position of the FAX-1 NRE is contacted by Ser-22 and Arg-79 of the DBD. The former amino acid residue is generally a Ser among NR2E3 subclass members and can be either a Ser or Ala among NR2E1 subclass members. Amino acid position 79, found in the T box, is a conserved arginine in all NR2E nuclear receptors. While the general conservation of protein-DNA contacts among nuclear receptors for which it has been directly tested is fairly good, we cannot be certain that other amino acids don't also participate directly in FAX-1-NRE binding. Furthermore, in all cases, other amino acids can contribute to DNA-binding specificity through conformation-specific contacts with the phosphodiester backbone [3,36].

The definition of potential DNA-binding sites allows us to predict candidate in vivo targets in the genome of *C. elegans*. There are 60 ARGTCA DR1 sites (assuming that the separating base-pair can vary) in the *C. elegans *genome. If we expand the list to include those sites that have a pyrimidine at the second position, ANGTCA DR1 sites, the number grows to 109 potential binding sites. These sites are not near any of the genes that are known to be dependent on *fax-1 *for their regulation (*flp-1, ncs-1, nmr-1, nmr-2*, and *opt-3 *[31]*)*. *fax-1 *may regulate these genes indirectly via another transcriptional regulator, by heterodimerization with another nuclear receptor that binds a different half-site, or by binding to other transcription factors at the promoter by a non-NRE-dependent mechanism. In addition, there are approximately 100 ARGTCA DR2 sites in the *C. elegans *genome, to which FAX-1 dimers may be able to bind *in vivo*.

Expanding the list to include those sites that allow FAX-1 dimeric binding via heteromeric sites that contain a weak site and a strong site (ANGTCANANNTCA or ANNTCANANGTCA) increases the number of potential FAX-1 binding sites in the genome to over 1200. The sequences upstream of *ncs-1 *and *opt-3*, both of which are regulated by *fax-1*, contain sites that match this more degenerate binding site (data not shown). With so many potential binding sites in the genome, other factors such as cognate coactivators or corepressors may help define the functional relevance of any particular candidate NRE. These observations suggest that members of the NR2E3 subclass are able to form dimers on a much wider variety of dimeric NRE's (over 1200), as compared to the more restrictive NR2E1 subclass (there are 36 AAGTCA DR1 sites). However, this does not mean that NR2E3 subclass members bind more sites *in vivo*; NR2E1 subclass monomers may also bind monomeric AAGTCA sites, of which there are over 20,000.

Studies on the human *fax-1 *ortholog, PNR, have demonstrated a similar DNA-binding activity [25,28]. PNR was found to bind to DR1 AAGTCA NRE's, but not to monomeric AAGTCA sequences. An unbiased screen for sequences that bind PNR revealed that, like FAX-1, it can bind sequences that vary at positions two and three of the half-site hexamer [28]. This study identified a preferred PNR binding site with the sequence AGRTCAAARRTCA, a sequence that is consistent with our analysis of FAX-1 DNA binding. While almost all binding sites revealed by this strategy include the TCA sequence at the 3' end of the half-sites, greater variability was observed for all three bases at the 5'end of the half-sites, including NRE position three in addition to NRE position two. Because Glu-19 of RAR and RXR directly contact NRE half-site position three in the RAR-RXR co-crystal, it may be that Asn-19 of FAX-1 and PNR also mediates the relaxed specificity at position three of the NRE. Therefore, the strong evolutionary conservation of the NR2E3 subclass DBD reflects, at least in part, the constraint of an evolutionarily-conserved NRE binding site.

Whether the DNA-binding activity that appears to be conserved between nematode and vertebrate members of the NR2E3 subclass translates into conserved patterns of gene regulation has yet to be investigated. PNR acts as a repressor of the transcription of cone-specific genes and may activate the transcription of rod-specific genes during vertebrate photoreceptor development [28,37]. The vertebrate orthologs of some of the targets of *fax-1 *regulation are also transcribed in vertebrate photoreceptor cells [31], which allows for the possibility of conserved gene regulatory patterns.

## Conclusion

Our results define subclass-specific DNA-binding specificities for the NR2E1 and NR2E3 subclasses, and suggest that binding preferences for the second nucleotide position of the DNA half-site are partially mediated by amino acid 19 of the NR2E DBD. The repertoire of potential dimeric binding sites is much larger for members of the NR2E3 subclass. FAX-1, like NR2E3 subclass member PNR, binds NRE's as a homodimer with a strong preference for DR1 sites. Therefore, dimerization behavior may be a second conserved difference between the NR2E1 subclass, members of which can bind as a monomer or homodimer, and the obligate dimer NR2E3 subclass.

## Methods

### Protein and nucleotide sequence analyses

We compared the amino acid sequences of nuclear receptors using the BLASTP 2.2.12 and CLUSTAL V programs [38,39]. The former was run with substitution matrix BLOSUM 80. The latter was run using Lasergene Megalign (DNAStar) with substitution matrix PAM 250. We performed alpha helix predictions for FAX-1 and NHR-67 using NNPREDICT [40] and PROF [41] and by comparing the results from these analyses to the known helical structures for related nuclear receptors [3,35]. We identified potential binding sites in genomic sequence using the BLASTN utility [38].

### Electrophoretic mobility shift assays

We performed EMSA using a procedure based on those described by Ausubel *et al.*[42] and Kobayashi *et al.*[25]. Oligonucleotides were synthesized and HPLC-purified by Invitrogen Corporation. The sequences of each oligo were complementary pairs (see Additional file 1). We expressed MBP::FAX-1 protein in *E. coli *strain ER 2508 as described previously [31] and purified MBP::FAX-1 protein via an amylase affinity column. In each experiment, we pre-incubated 1.5 μg of recombinant FAX-1 fusion protein for 30 minutes on ice in binding buffer (see Additional file 2). In competition experiments, we also added 4 ng, 40 ng, or 400 ng of cold competitor ds oligonucleotides. Following pre-incubation, we added 4 ng of ^32^P-labeled ds oligonucleotides and incubated for 30 minutes on ice, before loading onto a pre-run 8% non-denaturing polyacrylamide gel.

### One-hybrid experiments

We used the Clontech one-hybrid Matchmaker system to evaluate DNA-binding activity in yeast. The construction of *fax-1 DBD::GAL-4 AD, nhr-67 DBD::GAL4-AD*, and mutagenized derivate plasmids is described in Additional file 2. The portion of the FAX-1 protein (AAD55066) that was fused to the GAL4 activation domain is from Ala-95 to Asp-192. The NHR-67 construct fused residues Ile-15 through Gly-117 of the NHR-67 protein (NP 502094) to the GAL4 activation domain. We transformed and integrated the pGAD424-derived activation plasmids and pLacZi-derived reporter plasmids into yeast strain YM 4271 using the Li-acetate procedure described by the manufacturer. We assayed for β-galactosidase activity using a quantitative ONPG spectrophotometric assay, and calculated β-Gal units using the method of Miller [43]: 1000 × OD_420_/time of incubation × concentration factor × OD_600_.

## Competing interests

The author(s) declare that they have no competing interests.

## Authors' contributions

SC, RL, ELS, and SDD performed and analyzed one-hybrid experiments. SC, DRS, MC, and KR constructed and analyzed plasmids for one-hybrid experiments. BW conceived the project, developed it in concert with the other authors, performed the EMSA experiments, and drafted and revised the manuscript. All authors read and approved the final manuscript.

## Supplementary Material

Additional File 1 **Paired oligonucleotide sequences used for generating binding sites for EMSA and one-hybrid experiments**. Complete oligonucleotide sequences evaluated in this study. Click here for file

Additional File 2 **Supplementary methods**. Details of plasmid construction and experimental conditions for EMSA. Click here for file
