# Supplementary material for: Specificity of DNA-binding by the FAX-1 and NHR-67 nuclear receptors of Caenorhabditis elegans is partially mediated via a subclass-specific P-box residue
Source: BMC Mol Biol. 2008 Jan 7;9:2. doi: 10.1186/1471-2199-9-2 (PMC2225407; doi:10.1186/1471-2199-9-2)
Supplement: Additional File 1 — Paired oligonucleotide sequences used for generating binding sites for EMSA and one-hybrid experiments. Complete oligonucleotide sequences evaluated in this study. [file 1471-2199-9-2-S1.PDF]

PAIRED OLIGO SEQUENCES USED FOR GENERATING BINDING SITES FOR EMSA AND ONE-HYBRID EXPERIMENTS

DR1A
aattACCCTTTTAA**AAGTCA**A**AAGTCA**ACTTCCAA
TGGGAAAATTTTCAGTTTTCAGTTGAAGGTTagct

DR1G
aattACCCTTTTAA**AGGTCA**A**AGGTCA**ACTTCCAA
TGGGAAAATTTCCAGTTTCCAGTTGAAGGTTagct

DR1T
aattACCCTTTTAA**ATGTCA**A**ATGTCA**ACTTCCAA
TGGGAAAATTTACAGTTTACAGTTGAAGGTTagct

DR1C
aattACCCTTTTAA**ACGTCA**A**ACGTCA**ACTTCCAA
TGGGAAAATTTGCAGTTTGCAGTTGAAGGTTagct

DRNC
aattACCCTTTTAAAATTCAAAATTCAACTTCCAA
TGGGAAAATTTTAAGTTTTAAGTTGAAGGTTagct

MON1
aattACCCTTTTAA**AAGTCA**AAATTTAACTTCCAA
TGGGAAAATTTTCAGTTTTAAATTGAAGGTTagct

MON2
aattACCCTTTTAAAATTTAA**AAGTCA**ACTTCCAA
TGGGAAAATTTTAAATTTTCAGTTGAAGGTTagct

HRSW
aattACCCTTTTAA**AAGTCA**A**AATTCA**ACTTCCAA
TGGGAAAATTTTCAGTTTTAAGTTGAAGGTTagct

HRWS
aattACCCTTTTAA**AATTCA**A**AAGTCA**ACTTCCAA
TGGGAAAATTTTAAGTTTTCAGTTGAAGGTTagct
