# Supplementary material for: Specificity of DNA-binding by the FAX-1 and NHR-67 nuclear receptors of Caenorhabditis elegans is partially mediated via a subclass-specific P-box residue
Source: BMC Mol Biol. 2008 Jan 7;9:2. doi: 10.1186/1471-2199-9-2 (PMC2225407; doi:10.1186/1471-2199-9-2)
Supplement: Additional File 2 — Supplementary methods. Details of plasmid construction and experimental conditions for EMSA. [file 1471-2199-9-2-S2.PDF]

SUPPLEMENTARY METHODS

Electrophoretic Mobility Shift Assays

We annealed complementary oligonucleotide pairs by incubating a solution of 100 μM oligonucleotide (each) in 100 mM Tris-HCl, pH 7.5, 1 M NaCl and 10 mM EDTA to 65°C for 10 minutes, followed by slow cooling to room temperature. We confirmed that oligonucleotides were double-stranded by electrophoresis on a 5% MetaPhor agarose gel (Cambrex). We radioactively labeled double-stranded DNA by filling-in the 5' overhang at each end using DNA polymerase I Klenow fragment (Promega) and α-^{32}P-dATP (Amersham). We separated unincorporated nucleotide from labeled dsDNA using CENTRI-SEP spin columns as described by the manufacturer (Princeton Separations). The specific activity of labeled dsDNA, as defined in Sambrook et al., was greater than 9 x 10^8 cpm/μg.

We purified recombinant MBP::FAX-1 protein on an amylose affinity column as described by the manufacturer (New England Biolabs) and concentrated the protein using a Centricon 4M-30 spin column to a final concentration of 0.5 mg/ml. SDS-PAGE analysis of the recombinant product showed a major product at the expected molecular weight of about 80 kDa, and smaller products at about 65 kDa and 48 kDa (data not shown). We assume that the smaller products are due to proteolysis of full-length fusion protein since expression of the same plasmid in *E. coli* strain TB1, which expresses more functional proteases, showed a greater proportion of the smaller products.

EMSA experiments were performed in binding buffer: 20mM Hepes, pH 7.6, 30mM KCl, 1mM EDTA, 10 mM $(NH_4)_2SO_4$,1 mM DTT, 0.2% (w/v) Tween 20, 1 mM $MgCl_2$, 0.5 mg/ml bovine serum albumin, and 4ng, 40 ng, or 400 ng of cold competitor ds oligonucleotide (for competition experiments). Following pre-incubation, we added 4 ng of ^{32}P-labeled ds oligonucleotides and incubated for 30 minutes on ice, before loading onto a pre-run 8% non-denaturing polyacrylamide gel. We loaded each completed reaction onto a pre-run 8% non-denaturing polyacrylamide gel and ran the gel for 2 hours. We imaged each gel using a STORM 840 Phosphoimager and performed quantitative evaluation of each band using ImageQuant software on a Macintosh computer.

One-Hybrid Plasmid Constructions

To generate pGADFXCD1, we cloned the *C. elegans fax-1* cDNA sequences that correspond to the full DBD (including the CTE) into the yeast vector pGAD424 by digesting pFXCD5 with Alu I and Bam HI and ligating the resulting 290 bp fragment to pGAD424 that was digested with Xma I, filled in, and then digested with Bgl II. The portion of the FAX-1 protein (AAD55066) that was fused to the GAL4 activation domain is from Ala-95 to Asp-192. To create pGADN67, we digested *nhr-67* cDNA clone pN67CD2 with Eco RI, filled in the overhang, digested with Bam HI, and ligated the 308 bp fragment to the Sma I and Bgl II sites of pGAD424. The resulting clone fused NHR-67 (NP 502094) residues Ile-15 through Gly-117 to the GAL4 activation domain. We created mutagenized versions of the *fax-1*activation construct using a thermal cycling-mediated mutagenesis approach. We synthesized complementary oligonucleotides that changed the AAT Asn codon of FAX-1 into a GAT Asp codon as found in NHR-67. We

generated the DBD-containing fragment equivalent to the unmutagenized version by a combination of thermal cycling and primer extension with DNA polymerase I Klenow fragment. We generated the reciprocal change, which converts the GAT Asp codon of *nhr-67* into the AAT Asn codon of *fax-1*, using the Quick-Change mutagenesis system (Strategene). We confirmed the DNA sequence of the clones by dideoxy sequencing using the LiCor Automated DNA Sequencer at Muhlenberg College or the services of the University of Pennsylvania Sequencing Facility. We cloned candidate binding sequences into yeast vector pLacZi using the EcoRI and Xho I overhangs generated by the same double-stranded oligonucleotides used in the EMSA experiments. This puts the candidate binding site upstream of the minimal yeast promoter and *lacZ* coding region of pLacZi.

REFERENCE

Sambrook J, Russell DW: *Molecular Cloning: A Laboratory Manual.* Cold Spring Harbor, Cold Spring Harbor Laboratory Press 2001
